# Association between Vitamin D Receptor Polymorphisms (BsmI and FokI) and Glycemic Control among Patients with Type 2 Diabetes

**DOI:** 10.3390/ijerph18041595

**Published:** 2021-02-08

**Authors:** Wan Nur Amalina Zakaria, Nazihah Mohd Yunus, Najib Majdi Yaacob, Julia Omar, Wan Mohd Izani Wan Mohamed, K. N. S. Sirajudeen, Tuan Salwani Tuan Ismail

**Affiliations:** 1 Human Genome Centre, School of Medical Sciences, Universiti Sains Malaysia Health Campus, Kota Bharu 16150, Malaysia; dr_amalina@usm.my (W.N.A.Z.); nazihahmy@usm.my (N.M.Y.); 2 Unit of Biostatistics and Research Methodology, School of Medical Sciences, Universiti Sains Malaysia Health Campus, Kota Bharu 16150, Malaysia; najibmy@usm.my; 3 Department of Chemical Pathology, School of Medical Sciences, Universiti Sains Malaysia Health Campus, Kota Bharu 16150, Malaysia; juliakb@usm.my; 4 Department of Medicine, School of Medical Sciences, Universiti Sains Malaysia Health Campus, Kota Bharu 16150, Malaysia; izani@usm.my; 5 Department of Basic Medical Sciences, Kuliyyah of Medicine, International Islamic University Malaysia, Bandar Indera Mahkota, Kuantan 25200, Malaysia; knssiraj@iium.edu.my

**Keywords:** vitamin D receptor, type 2 diabetes mellitus, BsmI, FokI, glycemic control

## Abstract

(1) *Background:* Several studies have suggested that the vitamin D receptor (VDR) gene plays a role in type 2 diabetes mellitus (T2DM) susceptibility. Nonetheless, the association between T2DM and VDR polymorphisms remains inconclusive. We determined the genotype of VDR rs1544410 (BsmI) and rs2228570 (FokI) polymorphisms among Malaysian patients with T2DM and their association with glycemic control factors (vitamin D levels, calcium, magnesium, and phosphate). (2) *Methods:* A total of 189 participants comprising 126 patients with T2DM (63 with good glycemic control and 63 with poor glycemic control) and 63 healthy controls were enrolled in this case–control study. All biochemical assays were measured using spectrophotometric analysis. VDR gene FokI and BsmI polymorphisms were analyzed using polymerase chain reaction and endonuclease digestion. (3) *Results:* Our findings revealed no significant differences in VDR FokI and BsmI genotypes between participants with T2DM and healthy controls. Moreover, no significant association was observed between both single nucleotide polymorphisms and glycemic control factors. Participants with poor glycemic control had significantly lower serum magnesium levels and significantly higher HOMA-IR compared to the other groups. (4) *Conclusions:* The present study revealed that VDR gene BsmI and FokI polymorphisms were not significantly associated with T2DM.

## 1. Introduction

Approximately 50% of the population worldwide still suffers from vitamin D deficiency despite adequate sunlight exposure in Asian regions [1]. Accordingly, the prevalence of vitamin D deficiency has been attributed to weather, clothes, lifestyle, dietary intake, age, gender, predisposing of metabolic syndromes, and genetic heredity, all of which influence the bioavailability of vitamin D [2].

Accumulating evidence from human and animal studies has linked vitamin D status to insulin secretion and insulin resistance given that both vitamin D and its receptor complex play important roles in regulating the β-cell insulin secretion [3,4,5,6,7,8]. Furthermore, vitamin D deficiency has been associated with impaired insulin sensitivity, whereas vitamin D replacement in deficient individuals has been shown to improve insulin sensitivity [5,9,10]. Similarly, studies on animals and humans have shown that vitamin D receptor (VDR) knockout impaired glucose-induced insulin secretion, whereas vitamin D supplementation improved insulin secretory response [5,11,12].

VDRs, which are present in over 38 tissues, control vital genes related to bone metabolism, oxidative damage, chronic diseases, and inflammation [13]. The VDR gene, located on chromosome 12q13.1, consists of 14 exons (exons 2–9) and 6 untranslated exons (exons 1a–1f), with alternative splicing sites. Four common single nucleotide polymorphisms (SNPs) of the VDR gene are rs2228570 (FokI) in exon 2, rs1544410 (BsmI) and rs7975232 (ApaI) in intron 8, and rs731236 (TaqI) in exon 9 [14]. Earlier studies have shown that several VDR gene polymorphisms, such as BsmI and FokI, alter VDR protein activity [15,16]. All VDR polymorphisms are located between exons 8 and 9, except for FokI, which is located in exon 2 [17]. VDR polymorphisms are believed to be the primary reason for inherited VDR dysfunction.

The association between VDR polymorphisms and type 2 diabetes mellitus (T2DM) remains inconclusive. Studies conducted at various locations and involving a diverse group of individuals have observed various genetic VDR polymorphisms. To date, only one study in the central region of Malaysia has explored the association between the VDR BsmI (rs1544410) polymorphism and vitamin D deficiency, obesity, and insulin resistance among participants without diabetes across different age groups [18]. Accordingly, the aforementioned study had found that the BsmI (rs1544410) polymorphism was associated with increased risk for vitamin D deficiency and insulin resistance among the Malaysian population [18]. However, insufficient studies have investigated the effects of VDR polymorphisms on the modulation of glycemic control factors (i.e., vitamin D, calcium, magnesium, and phosphate levels) in Malaysian patients with T2DM. Among electrolytes abnormality, hypomagnesemia is the most frequently correlated with glycemic control in T2DM patients [19,20]. The current study therefore aimed to determine the possible association between VDR polymorphisms and diabetic phenotype, and obesity, among Malaysian patients with T2DM.

## 2. Materials and Methods

### 2.1. Study Participants

This case–control study recruited participants from the outpatient clinic and medical specialist clinic at Hospital Universiti Sains Malaysia (USM), Kelantan. Cases were defined as patients aged between 35 and 65 years old with confirmed T2DM based on the American Diabetes Association, 2015. Individuals presenting with factors that could potentially alter vitamin D metabolism, such as severe hepatocellular disease (e.g., liver cirrhosis), history of bone disease including recent fractures (within 6 months), chronic gastrointestinal disorder (loose stool or diarrhea for more than 3 months), gastric and small bowel resection, drugs that increase vitamin D metabolism (e.g., ritoglicazone, rifampicin, phenobarbital, and phenytoin), and vitamin D supplementation, were excluded from this study. Controls with similar age range as the cases were selected from relatives accompanying the patients and USM staff who volunteered. Body weight and height for all the participants were recorded and their Body Mass Index (BMI) were calculated to assess the obesity risk. They were categorized based on WHO guidelines into 18.5–24.9 as normal weight, 25–29.9 as overweight and ≥30 kg/m^2^ as obese. The study period was from February 2019 to February 2020 while the sample collection was performed from all participants in February till April 2019 that does not fall in the monsoon season.

### 2.2. Power Calculation

The largest sample size obtained was 57 participants with an F/f FokI genotype polymorphism proportion of 18.4% [21] among individuals with diabetes at a significance level (α) of 0.05 and precision of 0.1. After accounting for a dropout rate of 10%, the required sample size was 63 participants per group. Considering that analysis was to be conducted according to group, a total of 189 participants was required, among whom 63 had good glycemic control, 63 had poor glycemic control, and 63 had no diabetes.

Participants were classified into three main groups: (i) healthy controls (HbA1c less than 6.5% and random blood sugar less than 11.1 mmol/L), (ii) good glycemic control (good DM) (HbA1c less than or equal to 7.0% for at least two consecutive measurements or HbA1c less than 6.5% for a single measurement), and (iii) poor glycemic control (poor DM) (HbA1c more than 7.0% for at least two consecutive measurements).

### 2.3. Biochemical Measurements

A total of 10 mL of fasting venous blood samples was obtained from each study participant. Blood was collected into ethylenediaminetetraacetic acid tubes, plain bottles, and sodium fluoride tubes and centrifuged at 3500 rpm for 8 min at 25 °C. Thereafter, the collected plasma was aliquoted into microcentrifuge tubes (100 µL each). All blood and plasma samples were stored at −80 °C until assayed.

All biochemical assays, including fasting plasma glucose, serum calcium, serum magnesium, serum phosphate, and serum creatinine levels, were measured via spectrophotometric analysis using the ARCHITECT C800 analyzer (Abbott Diagnostics, Abbott Park, IL, USA). Serum 25(OH)D and fasting insulin levels were measured using the Elecsys^®^ Vitamin D total assay (Cobas, Roche Diagnostic Limited, Basel, Switzerland) and Elecsys^®^ Insulin kit (Roche Diagnostics Co., Indianapolis, IN, USA), respectively.

Vitamin D status was categorized into three groups according to World Health Organization and Institute of Medicine. Vitamin D deficiency, insufficiency, and sufficiency were defined as 25(OH)D levels <12 ng/mL (30 nmol/L), between 12–20 ng/mL (30–50 nmol/L), and ≥20 ng/mL (50 nmol/L), respectively [22,23]. Insulin resistance was calculated using the homeostatic model assessment of insulin resistance (HOMA-IR): FI (µU/mL) × FBG (mmol/L)/22.5. HOMA-IR value of more than 2.5 indicates insulin resistance in the general population [24].

### 2.4. Genotyping

Genomic DNA was extracted from the participants’ peripheral blood using the EXGENE Blood SV Mini Kit (GENEALL Biotechnology, Seoul, Korea) according to the manufacturer’s protocol with slight modification. The extracted DNA was used for amplifying target sequences of rs1544410 (BsmI) and rs2228570 (FokI) using sequence-specific primers. Primer sequences and conditions are presented in Table 1.

Polymerase chain reaction (PCR) amplification for rs1544410 (BsmI) and rs2228570 (FokI) was conducted separately in a 25 µL reaction mixture containing 2 µL of DNA sample, 0.5 µL of dNTP mix, 2 µL of MgCl, 1 µL of forward and reverse primers, 0.25 µL of Tag Polymerase, 5 µL of buffer, 0.25 µL of dimethyl sulfoxide, and 13 µL of double-distilled water. PCR cycling conditions consisted of initial pre-denaturation at 95 °C (2 min), denaturation at 95 °C (30 s), annealing (variable temperature based on SNPs), and extension at 72 °C for 30 s, followed by a final extension at 72 °C for 5 min. The process comprised of 35 cycles for each SNP. The amplified products were digested with MVA12691 (Bsm1) and Fast Digest Fok1 restriction enzymes at 37 °C, followed by genotyping on 3% agarose gel electrophoresis. Several randomly selected representative samples were sent for sequencing (First Base Labs, Kuala Lumpur, Malaysia), with the results being in concordance with RFLP genotyping.

### 2.5. Statistical Analysis

Data are expressed as means ± standard deviations. Normality of all numerical variables was assumed based on central limit theorem. Group comparisons for continuous and categorical variables were conducted using one-way analysis of variance with the Scheffe post hoc test and the Chi-square test, respectively. Haplotype analysis was performed using Haploview version 4.2 (Mark Daly’s lab at the Broad Institute of MIT and Harvard). Genotypic distribution was assessed for compatibility with Hardy-Weinberg equilibrium (HWE), with a *p*-value of more than 0.05 indicating agreement with HWE. Associations between genetic VDR polymorphism with insulin resistance (HOMA-IR), glycemic control factors (magnesium level, calcium level, phosphate level, vitamin D status), and obesity were analyzed using the Pearson correlation. Risk prediction was analyzed using simple and multiple logistic regression analyses. A *p*-value of less than 0.05 indicated statistical significance.

## 3. Results

### 3.1. Demographic and Biochemical Parameters

The majority of the study participants were Malay and female (133 out of 189). The healthy control group was significantly younger than the DM groups (*p* = 0.002). Participants with poor DM had significantly higher mean body mass index (BMI) compared to the healthy control group (*p* = 0.008). Participants with poor DM had significantly lower mean vitamin D level compared to those with good DM (*p* = 0.041). Among poor diabetic control and good diabetic control participants, 79% (50) and 36% (19) participants respectively have concomitant hypertension. However, overall systolic and diastolic blood pressure were less than 130 mmHg and 90 mmHg respectively in all participants. Systolic and diastolic blood pressure in good diabetic control were significantly lower than in poor diabetic control groups. Total cholesterol and low-density lipoprotein (LDL) in the poor diabetes control group were significantly higher than in the good diabetic control and healthy control groups. High-density lipoprotein (HDL) in the poor diabetes control group was low compared to the good diabetic control group, as expected. Surprisingly, the good diabetic control group had levels of triglyceride (TG) higher than the poor diabetic control group. The majority of the study participants had normal serum calcium, magnesium, and phosphate levels. Nevertheless, participants with poor DM had significantly lower serum magnesium levels (*p* < 0.001) and significantly higher HOMA-IR compared to healthy controls, with insulin resistance having been observed in 90.5% of those with poor DM, 84.1% of those with good DM, and only 58.7% of healthy control participants. Combination of insulin injection and oral hypoglycemic agent had been prescribed to 28% and 13% of participants in the poor and good diabetes control respectively (Table 2). Median IQR of duration of insulin usage among good diabetes control and poor diabetic control were 20 (25) and 12 (14) months, respectively. The vitamin D levels among healthy control, good and poor diabetic control participants in association with BMI categories are presented in Supplementary Tables S1–S4. Overall, the majority of obese participants had sufficient vitamin D levels.

### 3.2. Distribution of VDR 2228570 C > T (FokI) and VDR 1544410 G > A (BsmI) Gene Polymorphisms among Patients with T2DM Having Good and Poor Glycemic Control

The allele and genotype frequency distribution and carriage rate of VDR (FokI and BsmI) genes among patients with T2DM and healthy controls are summarized in Table 3. Among the participants with T2DM, the majority (53.2%) demonstrated the heterozygous CT genotype of the FokI polymorphism, 13.5% showed the variant TT genotype, and 33.3% had the homozygous wild-type CC genotype of the FokI polymorphism. Among the 63 healthy controls, 29 (46%) demonstrated the heterozygous CT genotype, 18 (28.6%) had the homozygous wild-type CC genotype, and 16 (25.4%) had the variant TT genotype of the FokI polymorphism.

Among the participants with T2DM, 73% had the homozygous wild-type GG genotype, 24.6% had the heterozygous GA genotype, and 2.4% had the variant AA genotype of the BsmI polymorphism. Among the control group, 71.4% showed the homozygous wild type GG genotype, 23.8% showed the heterozygous GA genotype, and 4.8% showed the variant AA genotype of the BsmI polymorphism. However, no significant differences in genotype and allele distributions of the VDR 2228570 C > T (FokI) and VDR 1544410 G > A (BsmI) polymorphisms were observed between participants with T2DM and healthy controls.

Among the participants with T2DM who had good and poor DM, the majority (54% and 52.4%) appeared to have the heterozygous CT genotype, 30.2% and 36.5% exhibited the homozygous wild-type CC genotype, and 15.9% and 11.1% had the variant TT genotype of the FokI polymorphism, respectively. However, no significant differences in genotype and allele frequencies of the FokI polymorphism were observed between participants with good and poor DM.

Likewise, no significant differences in genotype and allele distributions of the VDR 1544410 G > A (BsmI) polymorphism were observed between participants with good and poor DM. Homozygous wild-type GG genotype was predominant in both groups, followed by the heterozygous GA and variant AA genotypes in 4.8% of those with poor DM and none of those with good DM. Furthermore, we compared the VDR (FokI and BsmI) genotypes according to the different clinical parameters of all studied groups.

Genotypic distribution of VDR 2228570 C > T (FokI) and VDR 1544410 G > A (BsmI) were observed to be consistent with Hardy-Weinberg equilibrium in both cases and controls. Evaluation of linkage disequilibrium between FokI and BsmI based on *r*^2^ values showed that both SNPs were not in linkage disequilibrium (Supplementary Figure S1). Haplotype analysis showed no significant difference between the studied groups (Supplementary Tables S5 and S6).

### 3.3. Association between FokI (VDR 2228570 C > T) and BsmI (VDR 1544410 G > A) and Risk of Insulin Resistance

Table 4 shows the association between VDR 2228570 C > T (FokI) and VDR1544410 G > A (BsmI) polymorphisms and the risk of insulin resistance among healthy controls and participants with good DM. Accordingly, FokI and BsmI polymorphisms were found to have no association with the risk of insulin resistance among healthy controls and participants with good DM. Among healthy controls, those with BsmI, both heterozygous GA and variant AA genotypes, showed higher risk values (odds ratio (OR) 2.406, confidence interval (CI), 0.665–8.702 and OR 1.750, CI 0.148–20.707, respectively), although differences were not significant (*p* = 0.181 and 0.657, respectively).

The same analyses among participants with poor DM (Table 4) showed that the homozygous variant AA genotype of BsmI (VDR 1544410 G > A) was significantly associated with insulin resistance (*p* = 0.025), such that those with the AA genotype had a 95% lower likelihood of having insulin resistance. Meanwhile, no significant association was observed between FokI (VDR 2228570 C > T) and insulin resistance among participants with poor DM.

### 3.4. Association between FokI (VDR 2228570 C > T) and BsmI (VDR 1544410 G > A) and Glycemic Control Factors

Identical analyses were conducted to determine the association between VDR 2228570 C > T (FokI) and VDR1544410 G > A (BsmI) polymorphisms and glycemic control factors (vitamin D, calcium, magnesium, and phosphate levels) (Table 5, Table 6, Table 7 and Table 8). Accordingly, VDR 2228570 C > T (FokI) and VDR 1544410 G > A (BsmI) polymorphisms showed no significant association with all biochemical parameters in all groups.

### 3.5. Association between FokI (VDR 2228570 C > T) and BsmI (VDR 1544410 G > A) and Risk of Obesity

As shown in Table 9, a significant association was found between heterozygous CT genotype of FokI (VDR 2228570 C > T) and risk of obesity among healthy controls (*p* = 0.035), such that healthy participants with a heterozygous CT genotype had a 92% lower likelihood of becoming obese. However, no significant association was observed between BsmI (VDR 1544410 G > A) and risk of obesity among healthy controls (*p* > 0.05). Likewise, no significant association had been identified between VDR 2228570 C > T (FokI) and VDR 1544410 G > A (BsmI) polymorphisms and the risk of obesity among participants with good and poor DM.

## 4. Discussion

Studies have shown that vitamin D plays an essential role in insulin synthesis, secretion, and function, and elements of inflammation, which may affect the development of T2DM [25]. A meta-analysis of 11 studies by Shen et al. [26] found that patients with T2DM had lower vitamin D levels than controls, while Errouagui et al. [27] documented a higher prevalence of vitamin D deficiency among those with T2DM (40%) than among controls without diabetes (20%) in the Moroccan population. The current study found that among participants with T2DM, those having poor glycemic control exhibited significantly lower vitamin D levels compared to those having good glycemic control and healthy controls. Similarly, Mackawy and Badawi [28] revealed that Egyptian patients with diabetes, especially those with metabolic syndrome, had decreased vitamin D levels. The amount of vitamin D in obese and lean individuals may be similar, but the serum 25(OH)D level in obese individuals is usually lower because of the larger volume distribution in obese people [29]. Surprisingly in this study, the majority of obese participants had a sufficient amount of 25(OH)D. The probable reasons could be high dietary vitamin D intake, and longer duration of sun exposure and skin pigmentation in obese participants. These factors were not assessed in this study and the sampling among the obese was low, thus limiting the conclusiveness of the results.

Previous studies found that VDR gene polymorphisms affect VDR protein activity. Genetic variations in the VDR, which altered calcium metabolism, adipocyte function, insulin release, and cytokine expression, played a significant role in the pathogenesis of T2DM [9]. However, previous studies presented inconsistent, inconclusive, and variable results according to study populations and ethnic groups.

The present study evaluated the association between two potentially functional VDR gene variants (BsmI and FokI) and glycemic control factors among healthy controls and patients with T2DM who had good and poor glycemic control. Accordingly, our findings showed that neither VDR (FokI and BsmI) genotype was significantly associated with diabetes risk among the Malaysian population.

Moreover, haplotype analysis conducted herein showed no significant association between both SNPs and diabetes. This result was consistent with findings presented by Bid et al. [30] in the North Indian population and Malecki et al. [31] in the Polish population, both of whom found no correlation between VDR gene polymorphisms and diabetes at any of the four polymorphic sites. Likewise, a meta-analysis conducted by Yu et al. [32] concluded that no significant association existed between BsmI and FokI polymorphisms and T2DM. Nonetheless, results have varied according to sample size and study population ethnicity.

However, a study by Li et al. [21] among the Asian community revealed a possible link between polymorphisms at the FokI site and the onset of T2DM. Similarly, a study by El Gendy et al. [33] observed significant differences in FokI genotypes and allele distribution between Egyptian patients with T2DM and controls, which could be a risk factor for T2DM. Another study by Ortlepp et al. [34] observed a significant association between the BsmI VDR genotype and fasting glucose, while Oh and Barrett-Connor [35] observed a significant association between the VDR 1544410 (BsmI) polymorphism and HOMA-IR levels among individuals with T2DM in the Rancho Bernando Cohort.

The present study showed a significant association between insulin resistance and VDR 1544410 G > A (BsmI) polymorphism among patients with T2DM who had poor glycemic control. Moreover, our results showed that GG and GA genotype carriers had higher HOMA-IR levels compared to homozygous variant AA genotype carriers, suggesting that the homozygous variant AA genotype appeared to be protective against insulin resistance. Furthermore, we noticed significant associations between the VDR 2228570 C > T (FokI) polymorphism and risk of obesity among healthy participants without diabetes. Participants carrying the heterozygous CT genotype of the FokI polymorphism had lower BMI levels compared to those carrying the homozygous wild-type (CC) and variant (TT) genotypes.

These contradictory findings may be related to the divergent genetic backgrounds of the populations studied. Non-identical ethnic groups may have varying numbers of susceptibility alleles. T2DM has a complicated etiology involving polygenic heredity. Accordingly, various allele integrations may exist among patients with diabetes. Subsequently, abnormalities in insulin secretion associated with VDR polymorphisms might play an important role only in specific environmental or genetic backgrounds. Moreover, reported VDR polymorphisms may possibly be just markers of linkage disequilibrium with another gene, which may be responsible for the associations observed with type 2 diabetes mellitus. Nonetheless, more polymorphisms likely remain to be discovered [9].

Growing evidence has revealed that individuals with diabetes have impaired cellular calcium homeostasis. Accordingly, investigations on cellular calcium regulation defects in multiple cells, including cardiac muscle, skeletal muscle, kidneys, adipocytes, liver, osteoblasts, retinal tissue, and pancreatic beta cells, have confirmed that such defects are an underlying pathology associated with a diabetic state [36]. Hypocalcemia has been considered to be related to uncontrolled hyperglycemia among patients with T2DM, the correction of which may promote better glycemic control [37]. However, the present study found no significant association between calcium status and glycemic control given that none of our participants with our T2DM were hypocalcemic. The average calcium level observed among our study population may be attributed to the possible calcium-rich diet among our community, and increased physical activity considering that most of our study participants were younger than 60.

Hypophosphatemia has been generally associated with poor glycemic control. Accordingly, studies have shown that insulin, which increases the extracellular-to-intracellular transfer of phosphate, mediates the relationship between serum phosphate and glucose [38]. Moreover, hypophosphatemia has been implicated in the pathogenesis of diabetes mellitus, given that low serum phosphate levels promote insulin resistance and glucose intolerance. Considering the importance of phosphate in carbohydrate metabolism, reduced phosphate levels may decrease peripheral glucose use, leading to insulin resistance. The resulting compensatory hyperinsulinemia can further decrease phosphate concentrations, leading to the development of a vicious cycle that may contribute to the pathogenesis of metabolic syndrome [39]. Indeed, a previous study by De Fronzo and Lang agreed that chronic hypophosphatemia resulted in decreased tissue insulin sensitivity [40], while subsequent studies found that phosphorus supplementation for patients with hypophosphatemia who had glucose intolerance improved glucose tolerance [41,42]. Nonetheless, the current study found no significant mean difference in serum phosphate levels among our participants regardless their diabetic status.

Hypomagnesemia is the most frequent electrolyte abnormality among ambulatory patients with diabetes and is frequently observed among patients with diabetic ketoacidosis. The most critical factor for the onset of hypomagnesemia among patient with diabetes is glycosuria-induced excessive urinary magnesium loss. The clinical consequences of hypomagnesemia include impaired insulin secretion, insulin resistance, and increased macrovascular risk. However, the role of magnesium deficiency in microvascular complications has yet to be clearly established [43]. The aforementioned mechanism can explain our findings of hypomagnesemia only among participants with T2DM who had poor glycemic control [19]. None of the healthy controls and cases with good glycemic control included herein exhibited hypomagnesemia. Appropriate magnesium supplementation might prove beneficial for normalizing low plasma and tissue magnesium levels, subsequently preventing or hindering the development of vascular complications among patients with diabetes [44].

The current study found that among participants with T2DM, those with poor glycemic control had significantly higher BMI compared to those with good glycemic control and healthy controls. This is consistent with the findings of Daousi et al. [45], who reported that 86% of adults with T2DM were overweight or obese, with at least 52% having obesity and 8.1% having morbid obesity. Insulin resistance is one of the vital factors in the etiopathogenesis of T2DM. Accordingly, HOMA-IR and certain obesity indices have been identified as significant independent determinants of glucose intolerance. Indeed, a study by Lawal et al. [46] proposed the periodic use of HOMA-IR assessment on high-risk individuals, such as obese individuals and those whose first-degree relatives had diabetes, to identify those on the pathogenetic ladder toward glucose intolerance for early T2DM intervention. Our study observed that those with poor glycemic control had a significantly higher mean difference in HOMA-IR compared to those with good glycemic diabetic, with healthy controls having the lowest mean difference in HOMA-IR. Such findings are consistent with those presented in previous studies that suggested HOMA-IR as an established index of insulin resistance for the assessment of patients with T2DM [47].

One limitation of the current study is that our small population, which only included participants from the Kelantan region mainly consisting of the Malay community, may not be representative of the actual genetic polymorphisms among all Malaysians. Hence, more genetic epidemiological studies including larger populations ideally from all main ethnicities, including Malay, Chinese, and Indians, are required for a better understanding of the relationship between VDR variations and various phenotypes for insulin sensitivity, glycemic control factors, anthropometric data, and potential clinical implications.

Given the aforementioned findings, the current study concluded no significant association existed between VDR FokI and BsmI polymorphisms and T2DM. Nevertheless, our results suggested that the BsmI polymorphism was associated with insulin resistance among participants with T2DM who had poor glycemic control and that the VDR FokI polymorphism was associated with obesity risk among participants without diabetes. Moreover, those with poor DM had significantly lower serum magnesium levels and significantly higher HOMA-IR compared to the other two groups.

## Figures and Tables

**Table 1:** List of primers used for PCR amplification of regions containing the polymorphism and the restriction enzymes involved.

SNPid	Primers	PCR Product Size	Restriction Enzyme	Recognition Sequences
(rs1544410)	Fwd: CGGGGAGTATGAAGGACAAA Rev: CCATCTCTCAGGCTCCAAAG	348 bp (243 + 105 bp)	BSM1	5′…GAATGCN^▼^…3′ 3′…CTTAC_▲_GN…5′
(rs2228570)	Fwd: CTGGCACTGACTCTGGCTCT Rev: TATGACCTGTGAAGGCTGCA	183 bp (62 + 121 bp)	FOK1	5′…GGATG(N)_9_^▼^…3′ 3′…CCTAC(N)_13__▲_…5′

^▲^ and ^▼^ indicates cutting site of the restriction enzymes for its specific recognition sequences of nucleotides.

**Table 2:** Demographic and biochemical results.

Parameters	Healthy Control (*n* = 63)	Good DM Control (*n* = 63)	Poor DM Control (*n* = 63)	ANOVA (F-Test)	*p*-Value
Age (years)	50.33 ± 7.58	54.90 ± 7.77	53.14 ± 6.58	6.231	0.002
Gender					
-Male	11	25	20	-	0.022
-Female	52	38	43		
Ethnicity				-	0.211
-Malay	52 (82.5%)	58 (92%)	59 (93.6%)		
-Chinese	10 (15.9%)	5 (8%)	3 (4.8%)		
-Others	1 (1.6%)	-	1 (1.6%)		
BMI (kg/m^2^)	26.41 ± 4.5	27.87 ± 5.20	29.41 ± 5.912	5.048	0.007
BMI categories				-	0.135
Normal	25 (39.7%)	20 (31.7%)	18 (28.6%)		
Overweight	26 (41.3%)	26 (41.3%)	20 (31.7%)		
Obese	12 (19.0%)	17 (27.0%)	25 (39.7%)		
SBP (mmHg)	118.63 ± 5.85	121.67 ± 7.25	125.95 ± 9.39	14.61 (2, 186)	<0.001
DBP (mmHg)	78.71 ± 5.94	79.19 ± 5.95	82.54 ± 8.55	5.71 (2, 186)	0.004
TC	5.49 ± 0.79	5.87 ± 1.08	5.94 ± 0.81	4.46 (2, 186)	0.013
TG	0.96 ± 0.46	1.95 ± 2.22	1.20 ± 0.68	9.02 (2, 186)	<0.001
HDL	1.21 ± 0.33	1.30 ± 0.43	1.07 ± 0.27	6.40 (2, 186)	0.002
LDL	4.18 ± 0.80	3.75 ± 1.05	4.20 ± 0.80	5.10 (2, 186)	0.007
Vitamin D (ng/mL)	22.37 ± 8.81	25.48 ± 11.68	21.20 ± 8.33	3.256	0.041
Vitamin D categories				-	0.608
Sufficient	31 (49.2%)	38 (60.3%)	31 (49.2%)		
Insufficient	29 (46%)	22 (34.9%)	27 (42.9%)		
Deficient	3 (4.8%)	3 (4.8%)	5 (7.9%)		
Calcium (mmol/L)	2.28 ± 0.08	2.29 ± 0.09	2.33 ± 0.12	4.433	0.013
Magnesium (mmol/L)	0.92 ± 0.07	0.88 ± 0.07	0.81 ± 0.08	29.454	<0.001
Phosphate (mmol/L)	1.18 ± 0.19	1.17 ± 0.17	1.16 ± 0.18	0.292	0.747
HOMA-IR	3.69 ± 2.62	5.99 ± 3.69	19.62 ± 46.72	6.359	0.002
Insulin sensitive	26 (41.3%)	10 (15.9%)	6 (9.5%)	-	<0.001
Insulin resistant	37 (58.7%)	53 (84.1%)	57 (90.5%)		
Treatment	-			8.13 (1)	0.004
OHA alone		50 (79.4)	35 (55.6)		
OHA + Insulin		13 (20.6)	28 (44.4)		

**Table 3:** Genotype and allele frequency for VDR polymorphism at FokI (VDR 2228570 C > T) and BsmI (VDR1544410 G > A) among diabetic and healthy control.

Genotype	Healthy Control (*n* = 63)	Good DM (*n* = 63)	Poor DM (*n* = 63)	Healthy Control vs. DM		Good DM vs. Poor DM	
				OR (95% CI)	*p*-Value	OR (95% CI)	*p*-Value
**FokI (VDR 228570 C > T)**							
**Genotype**							
**CC**	18 (28.6%)	19 (30.2%)	23 (36.5%)	Reference		Reference	
**CT**	29 (46%)	34 (54%)	33 (52.4%)	0.990 (0.490–2.001)	0.978	0.802 (0.370–1.738)	0.576
**TT**	16 (25.4%)	10 (15.9%)	7 (11.1%)	0.455 (0.189–1.096)	0.079	0.578 (0.185–1.810)	0.347
**Allele**							
**C**	65 (51.6%)	72 (57.1%)	79 (62.7%)	Reference		Reference	
**T**	61 (48.4%)	54 (42.9%)	47 (37.3%)	0.713 (0.463–1.096)	0.123	0.793 (0.479–1.314)	0.369
**BsmI (VDR1544410 G > A)**							
**Genotype**							
**GG**	45 (71.4%)	48 (76.2%)	44 (69.8%)	Reference		Reference	
**GA**	15 (23.8%)	15 (23.8%)	16 (25.4%)	1.011 (0.496–2.060)	0.976	1.164 (0.515–2.628)	0.715
**AA**	3 (4.8%)	0	3 (4.8%)	0.489 (0.095–2.520)	0.393	N/A	
**Allele**							
**G**	106 (84.1%)	111 (88.1%)	105 (83.3%)	Reference		Reference	
**A**	20 (15.9%)	15 (11.9%)	21 (16.7%)	0.883 (0.488–1.600)	0.682	1.480 (0.725–3.023)	0.282

**Table 4:** Association between VDR polymorphism at FokI (VDR 2228570 C > T) and BsmI (VDR1544410 G > A) and risk of insulin resistance.

Group	FokI (VDR 2228570 C > T)					BsmI (VDR1544410 G > A)				
**Healthy control**	**Genotype**	**Insulin Sensitive (*n* = 26)**	**Insulin Resistance (*n* = 37)**	**OR (CI 95%)**	***p*-Value**	**Genotype**	**Insulin Sensitive (*n* = 26)**	**Insulin Resistance (*n* = 37)**	**OR (CI 95%)**	***p*-Value**
	CC	7 (26.9%)	11 (29.7%)	Reference		GG	21 (80.8%)	24 (64.9%)	Reference	
	CT	10 (38.5%)	19 (51.4%)	1.209 (0.358–4.089)	0.760	GA	4 (15.4%)	11 (29.7%)	2.406 (0.665–8.702)	0.181
	TT	9 (34.6%)	7 (18.9%)	0.495 (0.126–1.945)	0.314	AA	1 (3.8%)	2 (5.4%)	1.750 (0.148–20.707)	0.657
	Allele					Allele				
	C	24 (46.2%)	41 (55.4%)	Reference		G	47 (90.4%)	59 (79.7%)	Reference	
	T	28 (53.8%)	33 (44.6%)	0.690 (0.338–1.406)	0.307	A	5 (9.6%)	15 (20.3%)	2.390 (0.810–7.053)	0.115
**Good DM**	**Genotype**	**Insulin Sensitive (*n* = 10)**	**Insulin Resistance (*n* = 53)**	**OR (95% CI)**	***p*-Value**	**Genotype**	**Insulin Sensitive (*n* = 10)**	**Insulin Resistance (*n* = 53)**	**OR (95% CI)**	***p*-Value**
	CC	3 (30%)	16 (30.2%)	Reference		GG	6 (60%)	42 (79.2%)	Reference	
	CT	4 (40%)	30 (56.6%)	1.406 (0.280–7.072)	0.679	G	4 (40%)	11 (20.8%)	0.393 (0.094–1.640)	0.200
	TT	3 (30%)	7 (13.2%)	0.438 (0.070–2.728)	0.376	AA	0	0	N/A	
	Allele					Allele				
	C	10 (50%)	62 (58.5%)	Reference		G	16 (80%)	95 (89.6%)	Reference	
	T	10 (50%)	44 (41.5%)	0.710 (0.272–1.850)	0.483	A	4 (20%)	11 (10.4%)	0.463 (0.131–1.634)	0.232
**Poor DM**	**Genotype**	**Insulin Sensitive (*n* = 6)**	**Insulin Resistance (*n* = 57)**	**OR (95% CI)**	***p*-Value**	**Genotype**	**Insulin Sensitive (*n* = 6)**	**Insulin Resistance (*n* = 57)**	**OR (95% CI)**	***p*-Value**
	CC	2 (33.3%)	21 (36.8%)	Reference		GG	4 (66.7%)	40 (70.2%)	Reference	
	CT	3 (50%)	30 (52.6%)	0.952 (0.146–6.205)	0.959	GA	0 (0.0%)	16 (28.1%)	N/A	
	TT	1 (16.7%)	6 (10.5%)	0.571 (0.044–7.438)	0.669	AA	2 (33.3%)	1 (1.8%)	0.050 (0.004–0.681)	0.025
	Allele					Allele				
	C	6 (60%)	73 (62.9%)	Reference		G	6 (60%)	99 (85.3%)	Reference	
	T	4 (40%)	43 (37.1%)	0.884 (0.236–3.308)	0.854	A	4 (40%)	17 (14.7%)	0.258 (0.066–1.009)	0.052

**Table 5:** Association between VDR polymorphism at FokI (VDR 2228570 C > T) and BsmI (VDR1544410 G > A) and risk of vitamin D deficiency.

Group	FokI (VDR 2228570 C > T)					BsmI (VDR1544410 G > A)				
**Healthy control**	**Genotype**	**Vitamin D Sufficiency (*n* = 31)**	**Vitamin D Deficiency (*n* = 3)**	**OR (95% CI)**	***p*-Value**	**Genotype**	**Vitamin D Sufficiency (*n* = 31)**	**Vitamin D Deficiency (*n* = 3)**	**OR (95% CI)**	***p*-Value**
	CC	9 (29%)	1 (33.3%)	Reference		GG	25 (80.6%)	2 (66.7%)	Reference	
	CT	13 (41.9%)	1 (33.3%)	0.692 (0.038–12.572)	0.804	GA	5 (16.1%)	1 (33.3%)	2.500 (0.188–33.170)	0.487
	TT	9 (29%)	1 (33.3%)	1.000 (0.054–18.574)	>0.95	AA	1 (3.2%)	0	N/A	>0.95
	Allele					Allelle				
	C	31 (50%)	3 (50%)	Reference		G	56 (90.3%)	5 (83.3%)	Reference	
	T	31 (50%)	3 (50%)	1.000 (0.187–5.344)	>0.95	A	6 (9.7%)	1 (16.7%)	1.867 (0.186–18.734)	0.596
**Good DM**	**Genotype**	**Vitamin D Sufficiency (*n* = 38)**	**Vitamin D Deficiency (*n* = 3)**	**OR (95%CI)**	***p*-Value**	**Genotype**	**Vitamin D Sufficiency (*n* = 38)**	**Vitamin D Deficiency (*n* = 3)**	**OR (95%CI)**	***p*-Value**
	CC	13 (34.2%)	1 (33.3%)	Reference		GG	33 (86.8%)	2 (66.7%)	Reference	
	CT	20 (52.6%)	2 (66.7%)	1.300 (0.107–15.836)	0.837	GA	5 (13.2%)	1 (33.3%)	3.300 (0.251–43.470)	0.364
	TT	5 (13.2%)	0	N/A	0.999	AA	0	0	N/A	N/A
	Allele					Allele				
	C	46 (60.5%)	4 (66.7%)	Reference		G	71 (93.4%)	5 (83.3%)	Reference	
	T	30 (39.5%)	2 (33.3%)	0.767 (0.132–4.450)	0.767	A	5 (6.6%)	1 (16.7%)	2.840 (0.276–29.210)	0.380
**Poor DM**	**Genotype**	**Vitamin D Sufficiency (*n* = 31)**	**Vitamin D Deficiency (*n* = 6)**	**OR (95% CI)**	***p*-Value**	**Genotype**	**Vitamin D Sufficiency (*n* = 31)**	**Vitamin D Deficiency (*n* = 5)**	**OR (95% CI)**	***p*-Value**
	CC	13 (41.9%)	1 (16.7%)	Reference		GG	19 (61.3%)	4 (66.7%)	Reference	
	CT	14 (45.2%)	4 (66.7%)	3.714 (0.366–37.708)	0.267	GA	10 (32.3%)	1 (16.7%)	0.475 (0.047–4.839)	0.530
	TT	4 (12.9%)	1 (16.7%)	3.250 (0.163–64.614)	0.440	AA	2 (6.4%)	1 (16.7%)	2.375 (0.171–32.999)	0.519
	Allele					Allele				
	C	40 (64.5%)	6 (50%)	Reference		G	49 (79%)	9 (75%)	Reference	
	T	22 (35.5%)	6 (50%)	1.818 (0.523–6.317)	0.347	A	13 (21%)	3 (25%)	1.256 (0.297–5.317)	0.756

**Table 6:** Association between VDR polymorphism at FokI (VDR 2228570 C > T) and BsmI (VDR1544410 G > A) and risk of hypomagnesemia.

Group	FokI (VDR 2228570 C > T					BsmI (VDR1544410 G > A)				
**Healthy control**	**Genotype**	**Normal (*n* = 63)**	**Hypo Magnesemia (*n* = 0)**	**OR (95% CI)**	***p*-Value**	**Genotype**	**Normal (*n* = 63)**	**Hypo Magnesemia (*n* = 0)**	**OR (95% CI)**	***p*-Value**
	CC	18 (28.6%)	0	Reference		GG	45 (71.4%)	0	Reference	
	CT	29 (46%)	0	N/A	N/A	GA	15 (23.8%)	0	N/A	N/A
	TT	16 (25.4%)	0	N/A	N/A	AA	3 (4.8%)	0	N/A	N/A
	Allele					Allele				
	C	65 (51.6%)	0	Reference		G	106 (84.1%)	0	Reference	
	T	61 (48.4%)	0	N/A	N/A	A	20 (15.9%)	0	N/A	N/A
**Good DM**	**Genotype**	**Normal (*n* = 63)**	**Hypo Magnesemia (*n* = 0)**	**OR (95% CI)**	***p*-Value**	**Genotype**	**Normal (*n* = 63)**	**Hypo Magnesemia (*n* = 0)**	**OR (95% CI)**	***p*-Value**
	CC	19 (30.2%)	0	Reference		GG	48 (76.2%)	0	Reference	
	CT	34 (54%)	0	N/A	N/A	GA	15 (23.8%)	0	N/A	N/A
	TT	10 (15.9%)	0	N/A	N/A	AA	0 (0%)	0	N/A	N/A
	Allele					Allele				
	C	72 (57.1%)	0	Reference		G	111 (88.1%)	0	Reference	
	T	54 (42.9%)	0	N/A	N/A	A	15 (11.9%)	0	N/A	N/A
**Poor DM**	**Genotype**	**Normal (*n* = 50)**	**Hypo Magnesemia (*n* = 13)**	**OR (95% CI)**	***p*-Value**	**Genotype**	**Normal (*n* = 50)**	**Hypo Magnesemia (*n* = 13)**	**OR (95% CI)**	***p*-Value**
	CC	20 (40%)	3 (23.1%)	Reference		GG	34 (68%)	10 (76.9%)	Reference	
	CT	24 (48%)	9 (69.2%)	2.500 (0.595–10.500)	0.211	GA	14 (28%)	2 (15.4%)	0.486 (0.094–2.506)	0.388
	TT	6 (12%)	1 (7.7%)	1.111 (0.097–12.750)	0.933	AA	2 (4%)	1 (7.7%)	1.700 (0.139–20.749)	0.678
	Allele					Allele				
	C	64 (64%)	15 (57.7%)	Reference		G	83 (83%)	22 (84.6%)	Reference	
	T	36 (36%)	11 (42.3%)	1.304 (0.541–3.139)	0.554	A	17 (17%)	4 (15.4%)	0.888 (0.271–2.907)	0.844

**Table 7:** Association between VDR polymorphism at FokI (VDR 2228570 C > T) and BsmI (VDR1544410 G > A) and risk of hypocalcemia.

Group	FokI (VDR 2228570 C > T)					BsmI (VDR1544410 G > A)				
**Healthy control**	**Genotype**	**Normal (*n* = 62)**	**Hypocalcemia (*n* = 1)**	**OR (95% CI)**	***p*-Value**	**Genotype**	**Normal (*n* = 62)**	**Hypocalcemia (*n* = 1)**	**OR (95% CI)**	***p*-Value**
	CC	18 (29%)	0	Reference		GG	44 (71%)	1 (100%)	Reference	
	CT	29 (46.8%)	0	N/A	N/A	GA	15 (24.2%)	0	N/A	N/A
	TT	15 (24.2%)	1 (100%)	N/A	N/A	AA	3 (4.8%)	0	N/A	N/A
	Allele					Allele				
	C	65 (52.4%)	0	Reference		G	104 (83.9%)	2 (100%)	Reference	
	T	59 (47.6%)	2 (100%)	N/A	0.997	A	20 (16.1%)	0	N/A	0.998
**Good DM**	**Genotype**	**Normal (*n* = 63)**	**Hypocalcemia (*n* = 0)**	**OR (95% CI)**	***p*-Value**	**Genotype**	**Normal (*n* = 63)**	**Hypocalcemia (*n* = 0)**	**OR (95% CI)**	***p*-Value**
	CC	19 (30.2%)	0	Reference		GG	48 (76.2%)	0	Reference	
	CT	34 (54%)	0	N/A	N/A	GA	15 (23.8%)	0	N/A	N/A
	TT	10 (15.9%)	0	N/A	N/A	AA	0	0	N/A	N/A
	Allele					Allele				
	C	72 (57.1%)	0	Reference		G	111 (88.1%)	0	Reference	
	T	54 (42.9%)	0	N/A	N/A	A	15 (11.9%)	0	N/A	N/A
**Poor DM**	**Genotype**	**Normal (*n* = 63)**	**Hypocalcemia (*n* = 0)**	**OR (95% CI)**	***p*-Value**	**Genotype**	**Normal (*n* = 63)**	**Hypocalcemia (*n* = 0)**	**OR (95% CI)**	***p*-Value**
	CC	23 (36.5%)	0	Reference		GG	44 (69.8%)	0	Reference	
	CT	33 (52.4%)	0	N/A	N/A	GA	16 (25.4%)	0	N/A	N/A
	TT	7 (11%)	0	N/A	N/A	AA	3 (4.8%)	0	N/A	N/A
	Allele					Allele				
	C	79 (62.7%)	0	Reference		G	105 (83.3%)	0	Reference	
	T	47 (37.3%)	0	N/A	N/A	A	21 (16.7%)	0	N/A	N/A

**Table 8:** Association between VDR polymorphism at FokI (VDR 2228570 C > T) and BsmI (VDR1544410 G > A) and risk of hypophosphatemia.

Group	FokI (VDR 2228570 C > T)					BsmI (VDR1544410 G > A)				
**Healthy control**	**Genotype**	**Normal (*n* = 60)**	**Hypophosphatemia (*n* = 3)**	**OR (95%CI)**	***p*-Value**	**Genotype**	**Normal (*n* = 60)**	**Hypophos Phatemia (*n* = 3)**	**OR (95%CI)**	***p*-Value**
	CC	17 (28.3%)	1 (33.3%)	Reference		GG	44 (73.3%)	1 (33.3%)	Reference	
	CT	29 (48.3%)	0 (0%)	N/A	N/A	GA	13 (21.7%)	2 (66.7%)	6.769 (0.567–80.745)	0.131
	TT	14 (23.3%)	2 (66.7%)	2.429 (0.199–29.660)	0.487	AA	3 (5%)	0	N/A	N/A
	Allele					Allele				
	C	63 (52.5%)	2 (33.3%)	Reference		G	101 (84.2%)	5 (83.3%)	Reference	
	T	57 (47.5%)	4 (66.7%)	2.211 (0.390–12.529)	0.370	A	19 (15.8%)	1 (16.7%)	1.063 (0.118–9.617)	0.957
**Good DM**	**Genotype**	**Normal (*n* = 62)**	**Hypophosphatemia (*n* = 1)**	**OR (95% CI)**	***p*-Value**	**Genotype**	**Normal (*n* = 62)**	**Hypophos Phatemia (*n* = 1)**	**OR (95% CI)**	***p*-Value**
	CC	18 (29%)	1 (100%)	Reference		GG	47 (75.8%)	1 (100%)	Reference	
	CT	34 (54.8%)	0	N/A	0.998	GA	15 (24.2%)	0	N/A	0.999
	TT	10 (16.1%)	0	N/A	0.999	AA	0	0	N/A	N/A
	Allele					Allele				
	C	70 (56.5%)	2 (100%)	Reference		G	109 (87.9%)	2 (100%)	Reference	
	T	54 (43.5%)	0	N/A	0.997	A	15 (12.1%)	0	N/A	0.999
**Poor DM**	**Genotype**	**Normal (*n* = 61)**	**Hypophosphatemia (*n* = 2)**	**OR (95% CI)**	***p*-Value**	**Genotype**	**Normal (*n* = 61)**	**Hypophos Phatemia (*n* = 2)**	**OR (95% CI)**	***p*-Value**
	CC	23 (37.7%)	0 (0%)	Reference		GG	42 (68.9%)	2 (100%)	Reference	
	CT	32 (52.5%)	1 (50%)	N/A	0.998	GA	16 (26.2%)	0	N/A	N/A
	TT	6 (9.8%)	1 (50%)	N/A	0.998	AA	3 (4.9%)	0	N/A	N/A
	Allele					Allele				
	C	78 (63.9%)	1 (25%)	Reference		G	101 (82.8%)	4 (100%)	Reference	
	T	44 (36.1%)	3 (75%)	5.318 (0.537–52.682)	0.153	A	21 (17.2%)	0	N/A	0.998

**Table 9:** Association between VDR polymorphism at FokI (VDR 2228570 C > T) and BsmI (VDR1544410 G > A) and risk of obesity.

Group	FokI (VDR 2228570 C > T)					BsmI (VDR1544410 G > A)				
**Healthy control**	**Genotype**	**Normal (*n* = 25)**	**Obese (*n* = 12)**	**OR (95% CI)**	***p*-Value**	**Genotype**	**Normal (*n* = 25)**	**Obese (*n* = 12)**	**OR (95% CI)**	***p*-Value**
	CC	6 (24%)	5 (41.7%)	Reference		GG	19 (76%)	7 (58.3%)	Reference	
	CT	15 (60%)	1 (8.3%)	0.080 (0.008–0.836)	0.035	GA	5 (20%)	4 (33.3%)	2.171 (0.450–10.486)	0.334
	TT	4 (16%)	6 (50%)	1.800 (0.318–10.201)	0.507	AA	1 (4%)	1 (8.3%)	2.714 (0.149–49.533)	0.500
	Allele					Allele				
	C	27 (54%)	11 (45.8%)	Reference		G	43 (86%)	19 (79.2%)	Reference	
	T	23 (46%)	13 (54.2%)	1.387 (0.522–3.684)	0.511	A	7 (14%)	5 (20.8%)	1.617 (0.455–5.746)	0.458
**Good DM**	**Genotype**	**Normal (*n* = 20)**	**Obese (*n* = 17)**	**OR (95% CI)**	***p*-Value**	**Genotype**	**Normal (*n* = 20)**	**Obese (*n* = 17)**	**OR (95% CI)**	***p*-Value**
	CC	4 (20%)	5 (29.4%)	Reference		GG	16 (80%)	11 (64.7%)	Reference	
	CT	11 (55%)	10 (58.8%)	0.727 (0.151–3.493)	0.691	GA	4 (20%)	6 (35.3%)	2.182 (0.497–9.583)	0.301
	TT	5 (25%)	2 (11.8%)	0.320 (0.039–2.618)	0.288	AA	0	0	N/A	
	Allele					Allele				
	C	19 (47.5%)	20 (58.8%)	Reference		G	36 (90%)	28 (82.4%)	Reference	
	T	21 (52.5%)	14 (41.2%)	0.633 (0.252–1.594)	0.332	A	4 (10%)	6 (17.6%)	1.929 (0.496–7.500)	0.343
**Poor DM**	**Genotype**	**Normal (*n* = 18)**	**Obese (*n* = 25)**	**OR (95% CI)**	***p*-Value**	**Genotype**	**Normal (*n* = 18)**	**Obese (*n* = 25)**	**OR (95% CI)**	***p*-Value**
	CC	6 (33.3%)	10 (40%)	Reference		GG	12 (66.7%)	19 (76%)	Reference	
	CT	11(61.1%)	13 (52%)	0.709 (0.195–2.581)	0.602	GA	4 (22.2%)	6 (24%)	0.947 (0.221–4.067)	0.942
	TT	1 (5.6%)	2 (8%)	1.200 (0.089–16.239)	0.891	AA	2 (11.1%)	0 (0%)	N/A	0.999
	Allele					Allele				
	C	24 (66.7%)	32 (64%)	Reference		G	27 (75%)	45 (90%)	Reference	
	T	12 (33.3%)	18 (36%)	1.125 (0.456–2.773)	0.798	A	9 (25%)	5 (10%)	0.333 (0.101–1.099)	0.071

## Data Availability

The datasets generated and analyzed during the current study are available from the corresponding author on reasonable request.

## Supplementary Materials

The following are available online at https://www.mdpi.com/1660-4601/18/4/1595/s1, Tables S1–S4: The vitamin D levels among healthy control, good and poor diabetic control participants in association with BMI categories are presented, Tables S5 and S6: Haplotype analysis showed no significant differences between the studied groups, Figure S1: Linkage disequilibrium (LD) of FokI (VDR 2228570 C > T) and BsmI (VDR1544410 G > A).
