# Supplementary material for: Association between Vitamin D Receptor Polymorphisms (BsmI and FokI) and Glycemic Control among Patients with Type 2 Diabetes
Source: Int J Environ Res Public Health. 2021 Feb 8;18(4):1595. doi: 10.3390/ijerph18041595 (PMC7914454; doi:10.3390/ijerph18041595)
Supplement: Supplementary file 1 [file ijerph-18-01595-s001.pdf]

Supplementary Materials

Table S1. Vitamin D level among different category of BMI category in all study participants.

BMI Category	Vitamin D			Test Stat (df)	*p*-Value
	Sufficient (*n* = 100)	Insufficient (*n* = 77)	Deficiency (*n* = 12)		
Normal	37 (37.0)	23 (29.9)	3 (25.0)	-	0.848 [#]
Overweight	36 (36.0)	31 (40.3)	5 (41.7)		
Obese	27 (27.0)	23 (29.9)	4 (33.3)		

[#] Fisher exact test p-value.

Table S2. Vitamin D level among different category of BMI category in healthy control group.

BMI Category	Vitamin D			Test Stat (df)	*p*-Value
	Sufficient (*n* = 31)	Insufficient (*n* = 29)	Deficiency (*n* = 3)		
Normal	14 (45.2)	11 (37.9)	0 (0.0)	-	0.486 [#]
Overweight	11 (35.5)	12 (41.4)	3 (100.0)		
Obese	6 (19.4)	6 (20.7)	0 (0.0)		

[#] Fisher exact test p-value.

Table S3. Vitamin D level among different category of BMI category in good diabetic control.

BMI Category	Vitamin D			Test Stat (df)	*p*-Value
	Sufficient (*n* = 38)	Insufficient (*n* = 22)	Deficiency (*n* = 3)		
Normal	13 (34.2)	7 (31.8)	0 (0.0)	-	0.697 [#]
Overweight	16 (42.1)	9 (40.9)	1 (33.3)		
Obese	9 (23.7)	6 (27.3)	2 (66.7)		

[#] Fisher exact test p-value.

Table S4. Vitamin D level among different category of BMI category in poor diabetic control.

BMI Category	Vitamin D			Test Stat (df)	*p*-Value
	Sufficient (*n* = 31)	Insufficient (*n* = 26)	Deficiency (*n* = 6)		

Normal	10 (32.3)	5 (19.2)	3 (50.0)	-	0.613 [#]
Overweight	9 (29.0)	10 (38.5)	1 (16.7)		
Obese	12 (38.7)	11 (42.3)	2 (33.3)		

[#] Fisher exact test p-value.

Table S5. Haplotype frequencies of FokI (VDR 2228570 C > T) and BsmI (VDR1544410 G > A) among T2DM and healthy control.

rs1544410/ rs2228570	Frequency	DM (Cases)	Healthy Control	Chi-Square	*p*-Value
GC	0.487	0.509	0.441	1.556	0.2123
GT	0.360	0.344	0.392	0.846	0.3576
AC	0.085	0.090	0.074	0.254	0.6142
AT	0.069	0.057	0.092	1.621	0.2029

Table S6. Haplotype association of FokI (VDR 2228570 C > T) and BsmI (VDR1544410 G > A) among T2DM and healthy control.

rs1544410/ rs2228570	DM (Cases)	Healthy Control	OR (Confidence Interval)	*p*-Value
GC	128.4	55.6	(1) Reference	-
GT	86.6	49.4	0.759 (0.474–1.215)	0.251
AC	22.6	9.4	1.041 (0.457–2.371)	0.920
AT	14.4	11.6	0.538 (0.233–1.239)	0.141

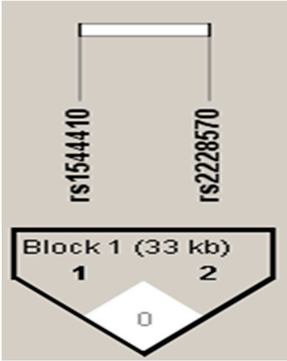

Figure S1. Linkage disequilibrium (LD) of FokI (VDR 2228570 C>T) and BsmI (VDR1544410 G>A).
